# Anterior Techniques in Managing Cervical Disc Disease

**DOI:** 10.7759/cureus.3146

**Published:** 2018-08-14

**Authors:** Lily H Kim, Marissa D'Souza, Allen L Ho, Arjun V Pendharkar, Eric S Sussman, Paymon Rezaii, Atman Desai

**Affiliations:** 1 Neurosurgery, Stanford University School of Medicine, Stanford, USA; 2 Neurosurgery, Stanford University School of Medicine, West Orange, USA

**Keywords:** cervical disc disease, anterior cervical discectomy, anterior cervical fusion, anterior cervical discectomy and fusion, acdf, stand-alone acdf, zero-profile spacer, total disc replacement, cervical disc arthroplasty

## Abstract

Surgical treatment may be indicated for select patients with cervical disc disease, whether it is cervical disc herniation or spondylosis due to degenerative changes, acute cervical injury due to trauma, or other underlying cervical pathology. Currently, there are various surgical techniques, including anterior, posterior, or combined approaches, in addition to new interventions being utilized in practice. Ideally, the surgical approach should be selected in consideration of each patient’s clinical presentation, imaging findings, and overall medical comorbidities on an individual basis. But the unique advantages and disadvantages of each surgical technique often complicate the therapy choice in managing cervical disc diseases. Although anterior cervical discectomy and fusion (ACDF) is the most widely accepted procedure performed for both single and multi-level cervical disc diseases, there are multiple modifications to this technique. Surgeons have access to different types of plates, screws, and cages and can adopt newer advances in the field such as stand-alone and minimally invasive techniques when indicated. In short, no consensus exists in terms of a single approach that is preferred for all patients. This article aims to review the standard of care for management of cervical disc disease with a focus on the surgical techniques and, in particular, the anterior approach, exploring the various surgical options within this technique.

## Introduction and background

For patients with cervical disc disease, most of which are due to cervical disc degeneration from natural aging, conservative treatment is still regarded as the first-line therapy. Oral neuropathic pain medications, physical therapy, and epidural steroid injections are the mainstays of nonsurgical approaches available for symptom management [1]. Although conservative treatment alone can be effective for many patients, surgery is indicated for those unresponsive to medical approaches or those with evidence of myelopathy with or without neurologic deficit. Surgical management of cervical disc disease may produce faster symptom resolution compared to conservative treatment. With the high prevalence of cervical disc disease that is only expected to rise with the aging population, surgical interventions have the potential to affect many patients experiencing significant morbidity. The ideal procedure should provide symptom relief and improved daily function while minimizing complications [2].

## Review

Anatomy

As one of the five regions of the spine, the cervical spine is made up of seven vertebrae. The most anterior and largest portion of the vertebrae is the body. Posteriorly, two pedicles and laminae form the vertebral arch. At this junction where the pedicle and lamina join each other is the transverse process, on each side of the vertebral body. Other processes of the vertebrae include spinous process in the midline and four articular processes. Two adjacent vertebrae are connected to each other by a ligamentous structure called intervertebral disc. Although this general vertebral anatomy applies throughout the cervical, thoracic, lumbar, sacral, and coccygeal spine, there are some unique properties to the cervical spine. First of all, the first two vertebrae have distinct names, atlas for C1 and axis for C2, reflecting their specialized structure and function. C1 (atlas), which lacks the body and spinous process, forms the atlanto-occipital joint as it articulates with the condyles of occipital bone, This synovial condylar joint is responsible for neck flexion and extension. C2 (axis) does have a vertebral body but it uniquely has a bony protuberance stabilized by the transverse ligament, called odontoid process, also known as the dens. Functionally, C2 plays an important role of supporting rotational movement of the neck. The rest of the cervical vertebrae, from C3 to C7, are similarly shaped and thus grouped together as the lower cervical spine. These five vertebrae house a vertebral body that is concave superiorly and convex inferiorly. They also have uncovertebral joints, Joints of Luschka, located between the uncinate process and uncus projecting from vertebral bodies of two adjacent levels. Overall, the flexibility of the cervical spine accommodates a wide range of motion but also increases its vulnerability to injury [1].

Anterior techniques

Ever since its first reported application by Smith and Robinson in 1955, the anterior approach has been considered a less invasive alternative to the traditional posterior technique [3]. Compared to the surgeries that access the cervical spine posteriorly, anterior approaches often yielded satisfactory results with less morbidity, minimal interference of the spinal canal, and superior fusion rate [4-5]. However, the evidence is mixed: patients who underwent anterior approaches tend to have better clinical outcomes than posterior approaches, but the difference was not always statistically significant, and the indications and specific pathology for each approach differ [6]. In other studies, anterior approaches were found to yield more approach-related complications such as dysphagia but result in a decreased incidence of reoperation.

Despite the lack of definitive advantage to either approach, both anterior and posterior techniques seem to have high success rates [7-8]. In general, the anterior approach is regarded a safe and effective option for patients with symptomatic cervical disc disease resulting from stenotic cervical canal of less than four levels. The anterior approach also allows for improved lateral decompression without destabilizing the posterior elements of the cervical spine. The posterior approach is less optimal than the anterior approach when patients exhibit a significant kyphosis, owing to the anatomical challenge in accessing the offending region and correcting kyphosis without accessing the disc space [1, 9-10].

The anterior technique consists of inducing spinal fusion by replacing the offending intervertebral disc with a spacer through dissection, discectomy, and grafting. Variations of this technique have been used in the field, including the Bailey-Badgley approach and Cloward approach. The latter essentially differs from the Smith-Robinson approach only by the orientation of graft alignment [11]. Surgeons can supplement discectomy with instrumentation or intervertebral cages to improve fusion and structural integrity of the end construct [5].

Discectomy With or Without Fusion

Anterior cervical discectomy (ACD) and anterior cervical discectomy with fusion (ACDF) are two different ways to perform anterior discectomy in cervical diseases. With ACDF, a spacer is inserted to facilitate fusion of the cervical spine. Studies have investigated the need for fusion by comparing ACDF with simple ACD, but to date, no Level I evidence definitively favors one method over the other for all patients [5]. However, multiple non-randomized prospective and retrospectives clinical series have suggested that discectomies without fusion, even when successfully done, are inferior to ACDF due to the increased likelihood of postoperative kyphosis and prolonged cervical pain [9]. Although Hauerberg et al. refuted this belief with their prospective randomized controlled trial showing insignificant difference between ACD and ACDF in terms of postoperative kyphotic deformity incidence [12], multiple studies suggest that patients who undergo ACD do have a greater chance of experiencing cervical kyphosis post operation whereas ACDF patients tend to maintain lordosis [13-14].

Kyphosis should not be the sole criteria to determine the superiority of one anterior technique over the other, especially since we still do not know the long-term consequences of kyphotic deformity [9]. Even when the risk of deformity is set aside, many different types of studies published over the last few decades, including a recent meta-analysis by Fraser and Hartl, point to ACDF as a superior option that is more likely to produce a more expedited and successful fusion, better functional outcome, and protection against disc space collapse [4, 13]. On the other hand, other randomized controlled trials deny that there is a significant difference in clinical outcome between patient populations treated with these two methods [8, 12]. These studies imply that both methods are effective in relieving cervical radiculopathy as long as it is arising from single compressed cervical nerve root level, adding emphasis on the importance of selecting the appropriate patient population in the surgical decision-making [14].

ACDF With or Without Instrumentation

ACDF can be performed with or without instrumentation. The advantages of instruments such as plates and screws include their ability to stabilize without external support and expedite fusion, especially in multi-level cases. At least in single-level cases, instrumentation has been reserved mainly for patients for whom the fusion rate is expected to be low, such as those with smoking history, osteoporosis, and advancing age [15]. Plating was not considered necessary when only one or two cervical discs were involved because the improved sagittal alignment after ACDF with instrumentation did not seem to affect clinical outcome compared to simple ACDF [16]. In fact, ACDF alone already boasts a high success rate that hovers around 90% regardless of instrumentation. But even in these cases, unplated patients have been shown to be more likely to require reoperation, which suggests that instrumentation may be effective in preventing poor outcomes such as pseudarthrosis or inadequate symptom relief that can lead to additional surgery [17-18].

There is also a concern that plated patients may experience injuries to soft tissues like the esophagus, postoperative dysphagia, or develop adjacent level disease in addition to requiring higher cost and lengthening operative time [19-20]. Acute trauma, reoperations, and history of adjacent segment fusion are strong indications for instrumentation. Smoking, nonsteroidal anti-inflammatory drug use, and menopause may also warrant the use of cervical plates due to increased rates of pseudarthrosis. Although a recent study suggested that postmenopausal women may not have a significantly increased risk of fusion failure compared to younger women [21], smoking remains to be one of the main predictors of unsuccessful fusion following spinal surgery [22].

Weighing the risks and benefits of plating has remained as one of the unresolved debates in cervical disc management. For instance, it is still unclear whether instrumentation itself is responsible for the degenerative changes of adjacent segments or if adjacent segment degeneration is part of the natural history of the disease. Chung et al. found that approximately 90% of patients who received ACDF with plating demonstrated some degree of degenerated adjacent segments, but when followed up for at least 10 years, most were not clinically significant nor required any additional management [23].

Hardware failure of the plate instrumentation is another point of contention in making the decision to employ instrumentation instead of performing ACDF alone. But the risk of hardware malfunction can be minimized with load-sharing dynamic plates, which have been in use since the late 1990s. Dynamic plates take advantage of the Wolff’s theory, which postulates that increased axial load-sharing promotes fusion. In a study conducted by Li et al., the incidence of hardware failure decreased significantly with the use of dynamic fixation instead of static plates [24].

In addition to dynamic plates, low-profile angle-stable plates and plates with smaller locking screws are some of the other new developments in fixation technique. Biodegradable plates and screws that can be resorbed are also a promising alternative with no significant long-term consequences to date. Although many have taken a conservative approach regarding plating, these newly developed plates have the potential to make fixation a more appealing option for a broader spectrum of patients [25].

Autograft Versus Allograft Versus Synthetic Materials

Grafting in ACDF entails inserting a graft to promote fusion of the operated region in the cervical spine. The grafts can be taken from the patient’s own body, usually the iliac crest bone (autograft), or from another person, often via the cadaveric bone bank (allograft). Though the two types of graft are both effective for fusion, neither of them are entirely free from complications. Though they have long been considered the gold standard, autografts are commonly associated with pain, infection, and fracture of the donor site. Allografts present with problems of recipient immune reaction, necrotic graft, and delayed fusion compared to autograft, often requiring the use of anterior cervical plates. Alternatives to autografts and allografts, such as ceramics, hydroxyapatite, and tricalcium phosphate, have gained a widespread use for their high biocompatibility and satisfactory ability to induce fusion [1].

Earlier in 2002, Kale and Cahill projected that intervertebral cage-dependent fusion may become a possibility for cervical disease management given that titanium and polymer cages are allowed for use in lumbar intervertebral fusion [5]. Since then, various types of intervertebral cages have been developed and utilized as cervical implants to avoid the adverse events associated with traditional grafts while maintaining a high fusion rate. Studies that have evaluated intervertebral cages showed that these devices can preserve disc height, facilitate the fusion process, and promote additional stability. Some of the currently available synthetic interbody cages include cages made of poly-ether-ether-ketone (PEEK), porous tantalum, and zero-profile anchored spacers. In a prospective, randomized trial by Chen et al., PEEK cages in multilevel cervical myelopathy were more likely to achieve favorable outcomes when compared to titanium cages in a long-term follow-up [26]. Studies have shown that low-profile PEEK cages may reduce esophageal damage because its placement is limited to the confinement of the intervertebral disc space. Other types of newer cages, such as hybrid cages that combine PEEK and titanium, need more clinical evidence to fully understand their efficacy and long-term safety [27].

Stand-Alone ACDF

Recently, a stand-alone ACDF technique has emerged in the field as an alternative to the standard plate and graft system. In this procedure, the aforementioned zero-profile, screw-anchored spacers are used instead of either the plate or cage as a separate entity [28]. The development of this newer system was fueled by the realization that use of plating systems are often followed by dysphagia and soft tissue damage [29-30]. By principle, a system that integrates the concept of inter-body spacer and plate in one device should enhance the unique benefits of plating and cage constructs while minimizing the complications [20, 31].

Indeed, multiple studies published in the last few years have demonstrated the significantly decreased rate of dysphagia, which can persist and negatively affect patients’ quality of life after ACDF [28, 32]. In one recent study of 104 patients, for instance, only 1.5% of the patients complained of dysphagia following stand-alone ACDF after 15 months of follow-up, significantly fewer in number than the 20% rate of dysphagia among patients who underwent the conventional ACDF with plating [19]. Adjacent segment disease also occurred less frequently with this approach, as can be expected from a device designed to have less rigid fixation of other cervical levels [33-34]. There were intraoperative advantages as well, with zero-profile spacers generally shortening operative time and decreasing blood loss [35-36].

Studies also report that this reduction in complications was achieved without compromising the overall surgical outcome [31]. The majority of patients had improved neck and arm pain and acceptable fusion rates that were comparable to the plate and graft system [37-39]. After reviewing 10 studies consisting of over 700 patients in their meta-analysis, Dong et al. concluded that zero-profile spacers conferred similar fusion rates, lower Neck Disability Index (NDI), and improved pain scores compared to standard anterior plating [28]. These favorable outcomes were replicated in both single level and multi-level cervical disc diseases, suggesting the safety of zero-profile spacers regardless of number of cervical levels involved [40, 41].

However, zero-profile spacers are not without potential drawbacks. For example, in a 2015 meta-analysis by Liu et al., this technique was associated with a higher rate of subsidence, although this risk did not lead to a clinical difference in disability or pain scores [34]. Moreover, it should be noted that most studies thus far have investigated the use of a particular stand-alone implant system called Zero-P® (DePuy Synthes, Raynham, MA), an angle-stable spacer approved for use by the United States Food and Drug Administration in 2008 [28, 42]. Some of the other implant options for stand-alone ACDF from different manufacturers include Coalition® (Globus Medical Inc., Audubon, PA), Optio-C® (Zimmer Spine, Minneapolis, MN), and Prevail® (Medtronic, Medtronic Sofamor Danek, Memphis, TN). Although the general function remains the same, these implants differ in the characteristics of the plate, screw, and cage that comprise the stand-alone system. The material of the plate is usually titanium or PEEK, and some implants like Zero-P® have additional features such as safety stop. Usually two to four screws are employed with nominal and variable angles specific to the implant system. Cage material is usually based on PEEK, but Zero-P® and Coalition® systems may use either PEEK or allograft [19].

Whether these technical differences among the implants carry clinical significance is yet to be seen. Not many studies investigated zero-profile anchored spacer systems other than Zero-P® and when they did, small sample sizes limited the generalizability of their findings [19-20]. Lack of long-term data, stemming from its relatively recent application in the field, is another challenge in assessing the full benefits or risks of stand-alone ACDF at present [20].

Minimally invasive surgeries

Expansion of minimally invasive approaches, namely micro-discectomy with the use of endoscopy is an increasingly popular approach. Cervical microendoscopic foraminotomy/discectomy (CMEF/D), cervical microendoscopic decompression of stenosis (CMEDS), and full endoscopic cervical discectomy (FECD) are some of the different methods that surgeons can employ to replace traditional discectomy [43]. Although these techniques are fairly new, a review of studies to date suggests that endoscopic procedures produce clinical outcomes comparable to traditional discectomy surgeries while lowering complication rates, patient discomfort, and hospitalization time. These techniques can also be applied to posterior surgeries. Given the limited scope of surgical field in minimally invasive approaches, expandable cages may be helpful in ensuring a stable graft positioning without the extensive retraction of nearby structures to gain a better access [7].

Anterior cervical foraminotomy is another less invasive, non-fusional alternative. This technique is gaining popularity, as it can effectively provide foraminal decompression for unilateral cervical radiculopathy while circumventing fusion-related complications that may be observed with ACDF. The procedural subtleties that are distinct from other anterior approaches may require additional operational exposure for surgeons to adopt this technique with proficiency [44].

Total disc replacement (cervical disc arthroplasty)

Total disc replacement in the cervical spine, also called cervical disc arthroplasty (CDA), is a surgical technique in which the offending disc is replaced with an artificial disc implant. CDA in many ways addresses problems of ACDF as a safe and an effective alternative that does not rely on fusion of adjacent segments. As many as one-fourth of patients who undergo ACDF go on to develop adjacent segment disease within 10 years of the procedure. The rates of adjacent segment disease following CDA is significantly lower, though not zero, with a recent meta-analysis by Zhu et al. estimating the risk to be almost halved by this newer approach (RR=0.57, p=0.009) [45]. In addition to development of adjacent segment diseases, the success of ACDF is also limited by frequent reports of diminished range of motion. CDA has been associated with superior cervical mobility with low rates of heterotopic ossification. The prosthetic devices approved for use at present include three that were initially approved: Bryan®  (Medtronic Sofamor Danek, Memphis, TN), Prodisc-C® (Synthes Spine, Paoli, PA), and Prestige LP® (Medtronic Sofamor Danek, Memphis, TN). Since 2012, three additional devices have been approved: Mobi-C® (Zimmer Spine, Minneapolis, MN), PCM® (NuVasive Inc., San Diego, CA), and Secure-C® (Globus Medical Inc., Audubon, PA). The data published to date seem to show acceptable durability and safety of devices with no major differences in the results depending on the type of prosthetics. In many cases, the selection of a particular type of implant is driven by the surgeon’s familiarity and preference [46].

Because CDA is still a relatively new technique, more long-term data would be needed for better assessment of its complications. The rate of heterotopic ossification following CDA is considered comparable to ACDF but this problem has been shown to increase with time after surgery. Moreover, implant longevity over a patient’s lifetime has not been studied due to lack of adequate time since the advent of currently available devices. Skovrlj and colleagues suggest that young patients who undergo CDA will most likely need a second surgery at some point following the initial intervention, which will inevitably carry risks associated with reoperation. Other less common but possible complications include device malfunction, prosthesis migration, and osteophyte formation [47].

Combination of anterior and posterior techniques

Anterior approaches have been widely used for their favorable long-term outcome with shortened hospitalization and reduced post-surgical pain when compared to the more traditional posterior approaches. However, posterior techniques may still provide unique benefits. For instance, posterior approaches can accommodate patients with exaggerated cervical lordosis, better stabilize disc space, and avoid interference of nearby structures. In fact, the superiority of either technique over the other has not been definitively established: Herkowitz et al. favored anterior approaches in 1990 whereas a more recent study by Dohrmann et al. concluded that posterior techniques resulted in a significantly higher long-term success rate (94% vs. 80%) [6, 48]. Because both the anterior and posterior techniques have their own advantages and disadvantages that often complement each other, a combined approach may be an option for select patients. One such combined procedure is microsurgical discectomy with laminoplasty, in use since its development by Fujimoto et al. in the early 2000s [49]. The outcome studies available to date indicate that combined approaches have similar success and complication rates compared to the traditional ACDF [1].

Developments in nonsurgical management

Management of cervical disc disease is quite successful at the present moment in the field. Surgeons have many options when it comes to choosing surgical techniques, most of which can produce a relatively fast symptomatic relief and also a safely maintained long-term clinical outcome. For instance, over 90% of patients who received an ACDF remained satisfied with the post-surgical outcome in a study that had a mean follow-up time of 6.5 years [11]. With newer technology in plating, intervertebral cages, and other lower-profile instrumentation in addition to development in minimally invasive techniques, management of cervical disc diseases will continue to improve.

In addition to the improvement seen within surgical modalities over the last several decades, there have been significant advances in medical management as well. These nonsurgical approaches can be used as a stand-alone treatment or as an adjunctive therapy either pre- or post surgery.

Cell-based therapies aim to use the regenerative capacity of mesenchymal stem cells and chondrocytes to reverse the degenerative process of the disc happening at the cervical level. Although mesenchymal stem cells have yet to show their efficacy in humans, autologous transplantation of chondrocytic cells performed after discectomy successfully improved pain in human studies. Due to the involvement of transplantation process, stem cell therapy may be considered surgical as well. Gene therapy and immunotherapy are still in their early stages of development but the promising in-vitro studies signal that we may soon be able to target specific genes and immune responses involved in disc degeneration. Tissue engineering is another such technique in development. These newer medical strategies differ from the existing conservative therapy in that they attempt to address the primary pathology instead of solely providing symptomatic relief [1, 50].

## Conclusions

Surgical management of cervical disc disease is safe and effective, achieving a significant reduction in patient morbidity with relatively small risks. Because current evidence points to multiple surgical approaches as being equally viable options, surgeons will need to be informed on the literature that evaluates different surgical techniques. Furthermore, in order to make the most beneficial and individualized treatment decision for each patient, study results should not be taken as an all-encompassing rule but as a general guideline to making a clinical decision.
